# Comparative evaluation of propolis mouthwash with 0.2% chlorhexidine mouthwash as an adjunct to mechanical therapy in improving the periodontitis among perimenopausal women: a randomized controlled trial

**DOI:** 10.1186/s12903-023-03768-4

**Published:** 2024-01-05

**Authors:** Syeda Maliha Waqar, Afifa Razi, Saima Sameer Qureshi, Fizza Saher, Syed Jaffar Abbas Zaidi, Chander Kumar

**Affiliations:** 1 Department of Oral Biology, Ziauddin College of Dentistry, Ziauddin University, Karachi, Pakistan; 2 Department of Oral Medicine and Diagnosis, Ziauddin College of Dentistry, Ziauddin University, Karachi, Pakistan; 3 Department of Periodontology, Ziauddin College of Dentistry, Ziauddin University, Karachi, Pakistan; 4 Department of Oral Biology, Dow Dental College, Dow University of Health Sciences, Karachi, Pakistan; 5 Department of Periodontology, Dow Dental College, Dow University of Health Sciences, Karachi, Pakistan

**Keywords:** Perimenopausal women, Propolis, Periodontal disease

## Abstract

**Objective:**

To evaluate the efficacy of Propolis mouthwash compared to chlorhexidine mouthwash as an adjunct to mechanical therapy in improving clinical parameters in perimenopausal women with chronic periodontitis.

**Methodology:**

A double-blind, randomized, controlled clinical trial was conducted by recruiting 144 subjects with mild to moderate chronic periodontitis. After scaling and root planning, subjects were allocated to two treatment groups: 0.2% chlorhexidine mouthwash and 20% propolis mouthwash twice daily for six weeks. Clinical parameters such as pocket probing depth (PPD), clinical attachment loss (CAL) and bleeding on probing (BOP) were analysed at baseline, six weeks, and 12 weeks.

**Result:**

The mean value of PPD in the propolis group was 4.67 at baseline, reduced to 4.01 at six weeks and 3.59 at 12 weeks. While in the chlorhexidine group, the baseline value of 4.65 reduced to 4.44 and 4.25 at six weeks and 12 weeks, respectively. The baseline value of the mean CAL in the propolis group was 4.45. This value was reduced to 4.15 at six weeks and 3.77 at 12 weeks. For the chlorhexidine group, the baseline value of CAL was 4.80, which was reduced to 4.50 and 4.19 at six weeks and 12 weeks. The mean value of bleeding on probing in the propolis group was 77.20, which decreased to 46.30 at six weeks and 14.60 at the final visit. In the chlorhexidine group, the mean value of 77.30 was reduced to 49.60 and 22.80 at subsequent visits.

**Conclusion:**

This study concludes that both propolis and chlorhexidine mouthwash positively improve clinical parameters; however, propolis is significantly more effective in improving BOP.

**Trial registration:**

ID: NCT05870059, Date of Registration: 02/02/2022. (https://beta.clinicaltrials.gov/study/NCT05870059).

## Introduction

Our oral cavity is the window into our overall health and well-being and harbours one of the most diverse microbial communities, from which more than 700 bacterial species can be isolated [1]. Several microbes in this group exhibit strong associations with periodontal disease, such as *Porphyromonas gingivalis, Tannerella forsythia, and Treponema denticola.* They are known as the Red Complex due to their high pathogenicity [2]. Periodontitis is a chronic inflammatory disease initiated by the periodontopathic bacterium in dental plaque, which produces pro-inflammatory mediators and activates the local immune system in response. This affects the supporting tissues around the teeth, resulting in progressive loss of the attachment apparatus and bone surrounding the teeth [3].

According to the global burden of disease study 2016, it is the 11^th^ most prevalent disease globally [4] and the 2^nd^ most common disease of the oral cavity following dental decay [5]. Periodontitis is prevalent in the adult population, and age is one of the most significant risk factors. At the age of 60 years and older, the prevalence is reported to be 93% [6].

Mechanical debridement through scaling and root planning (SRP) is the initial treatment approach for periodontal pockets [7]. However, in areas such as deep pockets where instruments are inaccessible, SRP alone cannot eliminate pathogenic bacteria, and antimicrobial mouthwash for chemical plaque control is accepted as an ideal vehicle [8]. In several clinical studies, 0.2% chlorhexidine mouthwash was demonstrated to be effective at preventing gingivitis [9–11]. Unfortunately, this gold standard antiseptic has some drawbacks, such as staining on teeth and discoloured restorations, unpleasant taste, and altered taste sensation [9]. The World Health Organization (WHO) has reported that approximately 4 billion people currently prefer herbal medicine as a remedy for their ailments these days [12]. A multi-ethnic group study found that 50% of Asians consume herbal products to maintain their health [13].

The use of herbal remedies such as Aloe Vera, black cumin oil and clove oil has been documented for treating periodontal disease [14, 15]. Propolis, also known as bee glue, is a natural resinous product with an incredibly wide range of therapeutic properties, including anti-inflammatory, antimicrobial, immunomodulatory, antitumor, anticancer, antiulcer, hepatoprotective, cardioprotective, and neuroprotective effects [16]. Standardizing the benefits has been difficult due to the complex chemical composition, since it significantly depends on the environment, geographic location, type of plant pollen, and bee species. Consequently, propolis collected from different regions of the world has distinct biological properties [17]. The primary biologically active ingredient responsible for the anti-inflammatory properties of the sample is caffeic acid phenyl ester (CAPE), which inhibit the lipoxygenase and cyclooxygenase enzymes and halts the arachidonic acid pathway [18]. Arachidonic acid cessation prevents the production of prostaglandins and leukotrienes responsible for pain and inflammation. CAPE also reduces the infiltration of neutrophils and monocytes and enhances the production of Interleukin 4 and Interleukin 10, which are anti-inflammatory cytokines [19]. However, direct comparative studies on the efficacy of Propolis and chlorhexidine mouthwashes as adjunctive therapies to mechanical periodontal treatment in perimenopausal women with chronic periodontitis are scarce. The lack of standardization and the limited understanding of the clinical effects of propolis, particularly in comparison to established treatments like chlorhexidine, pose significant gaps in managing periodontal disease.

The active component of propolis, caffeic acid phenyl ester (CAPE), exhibits anti-inflammatory actions by inhibiting pro-inflammatory pathways and promoting anti-inflammatory cytokines [20]. Hence, a comparative study between chlorhexidine and Propolis mouthwashes in managing the periodontitis could significantly contribute to existing knowledge and potentially transform the standard of care.

Therefore, this study aims to assess and compare the anti-inflammatory potential of 0.2% chlorhexidine mouthwash and 20% Propolis mouthwash as an adjunct to mechanical therapy (SRP) in improving the clinical parameters of chronic periodontitis.

## Methodology

### Study design and ethical considerations

This double-blind, randomized, controlled clinical trial was conducted in the Department of Oral Medicine and the Diagnosis and Department of Periodontology, Ziauddin College of Dentistry, and the Department of Periodontology, Dow Dental College. Ethical clearance was obtained from Ziauddin University **(Protocol number: 4300921MWOM)**. The collected propolis was authenticated by the Department of Pharmacognosy, Faculty of Pharmacy, University of Karachi, Karachi. **Voucher specimen No. A00179**. The study was also registered at www.clinicaltrials.gov with identifier no. **NCT05870059** on 02/02/2023.

### Patients recruitment

The sample size for this trial was calculated by the OpenEpi calculator is 51 for each group keeping a 95% confidence interval and 80% power of the test, which makes total of 102 for both groups [8]. To achieve this sample size, 254 women aged 40–50 were evaluated for eligibility criteria and 144 were recruited with chronic periodontitis from the outpatient department for ten months from December 2021 to May 2022. Before the recruitment, the procedure was explained and informed written consent was obtained. The oral examination of patients was performed as per the protocol described by the British Society of Periodontology, and the participants with PPD of 4–5 and CAL 1–4 were recruited in the trial, which corresponds to stage I and stage II according to 2017 classification of disease by American Association of Periodontology. Females with chronic periodontitis were further assessed for eligibility considering the following criteria. In this study, specific inclusion and exclusion criteria were employed to select subjects to ensure the integrity of the study.

**Fig. 1:**
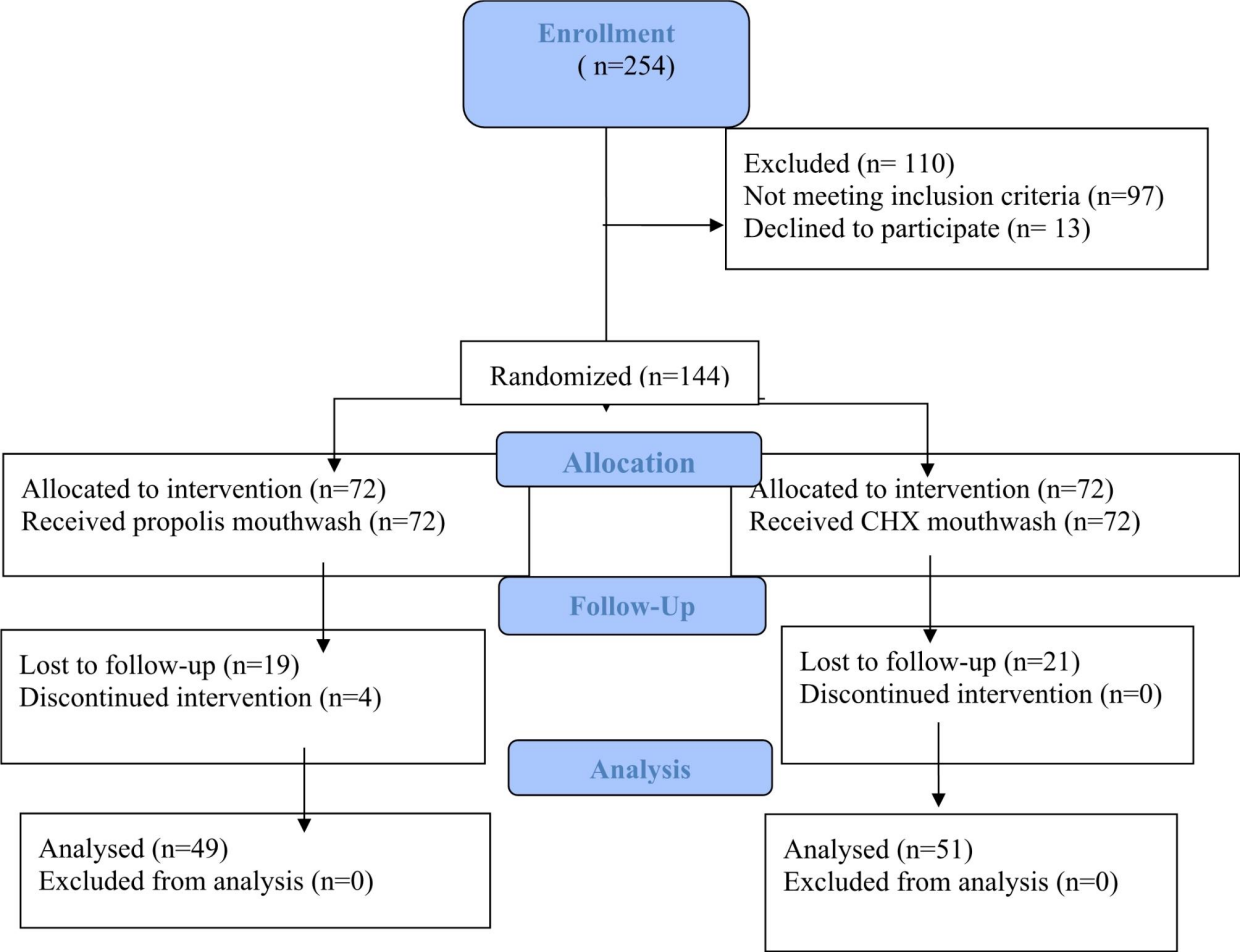
Consort flowchart of the study samples

For the inclusion criteria, the study focused on premenopausal female subjects ranging from 40 to 50 years of age. The menstrual history comprised of inquiries about the duration of menstrual periods and the average number of days between two cycles. This information is essential in monitoring the onset of irregularities and assessing the peri-menopausal stage. Only those patients who provided informed consent were included in the study as shown in Fig. 1. All selected patients were those with a probing pocket depth (PPD) between 4 and 5 mm and a clinical attachment loss (CAL) between 1 and 4 mm. Only those patients were included that displayed bleeding on probing (BOP). To control confounding factors, the study excluded subjects who had undergone periodontal therapy or taken antibiotics six months before the study. Additionally, subjects were required to have a minimum of 20 teeth in their oral cavity. Patients with known allergies to honey products were excluded from the study, as these individuals might have adverse reactions that would affect the study outcome. Patients who had lost teeth due to periodontal disease were also excluded, as their dental health was already compromised. The detailed CONSORT flow chart is shown in Fig. 1.

Additionally, individuals who are current smokers or have been smokers in the past were not considered for this study to prevent the confounding effects of tobacco on periodontal health. Another exclusion criterion excluded patients on any form of antibiotic therapy during the screening period. Lastly, patients with systemic conditions that predispose them to chronic periodontal disease, such as diabetes, were also not included in this study. This exclusion ensured that the study was focused on patients whose secondary systemic condition did not influence periodontal disease.

### Dispensing and distribution of mouthwash

In compliance with the double-blind study protocol, an independent third party was assigned the task of dispensing both Propolis mouthwash, formulated at Ziauddin College of Pharmacy, and commercially available chlorhexidine mouthwash into identical, opaque-colored bottles. This third party then labelled the bottles as either ‘A’ or ‘B’ and sealed the corresponding codes along with the product names in separate envelopes, which were to be unveiled post-trial. A bottle of each group (A and B) was presented to patients for randomization. The patients were allowed to independently select one of the two bottles provided, eliminating any bias or influence in the choice of mouthwash.

### Group distribution and intervention

We divided 144 participants into two equal groups of 72 subjects, and the treatment advised was:

- Group I: (n = 72) 20% Propolis Mouthwash twice daily for six weeks.
- Group II: (n = 72) 0.2% chlorhexidine Mouthwash twice daily for six weeks.

Before allocating the subjects into different groups, all participants underwent the standard scaling and root planning treatment and were instructed on standard oral hygiene measures such as toothbrushing and flossing followed by mouthwash rinse.

A checklist was provided to ensure the compliance with the experimental and control mouthwashes and constant reminders were provided on call. They were also advised to bring the empty mouthwash bottle to the follow-up appointment.

### Patient assessment

A single calibrated examiner assessed treatment outcomes through full mouth probing at baseline, six weeks, and 12 weeks using the Williams periodontal probe.

The following indices were measured to assess the progress of periodontal disease:

I) Periodontal pocket depth (PPD).
II) Clinical attachment loss (CAL).
III) Bleeding on probing (BOP).

### Statistical analysis

Data were analysed using the statistical package of social sciences (SPSS) software version 23. Quantitative data was analysed by mean and standard deviation. Repeated measures of ANOVA were applied to see the treatment outcome.

## Results

No significant differences between the two groups were found in age, as it is 45.69 ± 0.47 in the propolis group and 45.35 ± 0.51 in the CHX group.

The mean value of PPD in the propolis group was 4.67 at baseline, which reduced to 4.01 at six weeks and 3.59 at 12 weeks. While in the chlorhexidine group, the baseline value of 4.65 reduced to 4.44 and 4.25 at six weeks and 12 weeks, respectively.

A statistically significant difference between the groups was observed in PPD at six weeks (p = 0.001) and 12 weeks (p = 0.001). When PPD was compared within groups, a statistically significant decrease in PPD at six weeks and 12 weeks was observed from the baseline in both groups (p < 0.05). (Table 1)

**Table 1:** Comparison of PPD at baseline, six weeks, and 12 weeks between groups and within groups

	Groups		p-value
	Propolis (n = 49)	CHX (n = 51)	
**Baseline**	4.67 mm (4.56–4.89)	4.65 mm (4.435–4.89)	0.548
**6 weeks**	4.01 mm (3.72–4.155)	4.44 mm (4.16–4.63)	0.001*
**12 weeks**	3.59 mm (3.28–3.92)	4.25 mm (4.02–4.48)	0.001*
**p-value**	0.001*	0.001*	

*Significant at 5% level of significance

*Mann whitney U test; *Friedmans

The baseline value of CAL in the propolis group was 4.45. That was reduced to 4.15 at six weeks and 3.77 at 12 weeks. For the chlorhexidine group, the baseline value of 4.80 was reduced to 4.50 and 4.19 at 6 and 12 weeks. A statistically significant difference was observed in CAL at baseline (p = 0.017), six weeks (p = 0.003) and 12 weeks (p = 0.001) between groups.

When CAL was compared within groups, a statistically significant decrease in CAL was observed at six weeks and 12 weeks from the baseline in both groups (p < 0.05). (Table 2)

**Table 2:** Comparison of CAL at baseline, six weeks, and 12 weeks between groups and within groups

	Groups		p-value
	**Propolis (n = 49)**	**CHX (n = 51)**	
**Baseline**	4.45 ± 0.73 mm	4.80 ± 0.7 mm	0.017*
**6 weeks**	4.15 ± 0.57 mm	4.50 ± 0.61 mm	0.003*
**12 weeks**	3.77 ± 0.51 mm	4.19 ± 0.56 mm	0.001*
**p-value**	0.001*	0.001*	

*Significant at 5% level of significance

Independent sample t test * ANOVA

The mean value of bleeding on probing in the propolis group was 77.20, which decreased to 46.30 at six weeks and 14.60 at the final visit. In the chlorhexidine group, the mean value of 77.30 was reduced to 49.60 and 22.80 at later visits. A statistically significant difference between these groups was observed in BOP at six weeks (p = 0.017) and 12 weeks (p = 0.001). However, the improvement in BOP in the propolis group was slightly greater than that of chlorhexidine. When BOP was compared within groups, a statistically significant decrease in BOP was observed at six weeks and 12 weeks from the baseline (p < 0.05) (Table 3). The distribution between the groups was normal and owing to the differences in baseline values, independent sample t-test was used to compared between the propolis and the chlorhexidine group. Furthermore, we have addressed baseline differences in this study through randomization. The process of randomly assigning participants to treatment groups helps ensure that any potential biases due to baseline differences are evenly distributed across the groups.

**Table 3:** Comparison of BOP at baseline, six weeks, and 12 weeks between groups and within groups

	Groups		p-value
	Propolis (n = 49)	CHX (n = 51)	
**Baseline**	77.20 mm (71.40-89.39)	77.30 mm (68.65–86.50)	0.578
**6 weeks**	46.30 mm (40.97–51.32)	49.60 mm (43.05–55.85)	0.017*
**12 weeks**	14.60 mm (12.60–16.80)	22.80 mm (18.80–26.50)	0.001*
**p-value**	0.001*	0.001*	

*Significant at 5% level of significance

*Mann whitney U test; *Friedmans test

## Discussion

Over the past decade, propolis has gained recognition in pharmaceuticals and nutraceuticals for its therapeutic properties in the treatment of tumours, inflammatory conditions, bacterial infections, and parasitic infections. Recent experimental evidence regarding the anti-inflammatory mechanism of propolis was reviewed by Zulhendri F. et al. and concluded that it is due to the downregulation of TLR4, MyD88, IRAK4, TRIF, NLRP inflammasomes, NF-κB, and their associated pro-inflammatory cytokines such as IL-1β, IL-6, IFN-γ, and TNF-α that makes propolis an anti-inflammatory agent. Propolis also downregulates the chemokines such as CXCL9 and CXCL10 and reduces the migration of immune cells such as macrophages and neutrophils [21, 22].

The present study investigated the comparative efficacy of Propolis and chlorhexidine mouthwashes as adjunctive therapy to mechanical periodontal treatment in perimenopausal women with chronic periodontitis. The results indicate that both types of mouthwash demonstrated positive outcomes, with Propolis showing a significantly more pronounced effect on bleeding on probing (BOP).

Clinical parameters, such as pocket probing depth (PPD), clinical attachment loss (CAL), and BOP, showed significant improvement in both groups across the 12-week study duration. However, the Propolis group demonstrated a significant decrease in all parameters, particularly in BOP, suggesting a superior anti-inflammatory effect. This finding adds to the existing literature in which a significant reduction in the papillary bleeding index was observed after using Propolis mouthwash compared to placebo in two groups (n = 16) on the 15^th^ and 30^th^ day after treatment [23].

### Periodontal pocket depth (PPD)

In this study, the reduction in PPD is a significant finding in both the Propolis and chlorhexidine groups. The reduction in PPD in the Propolis group is noteworthy, starting from a baseline value of 4.67 mm, which decreased to 4.01 mm at six weeks and further to 3.59 mm at 12 weeks. The chlorhexidine group also experienced a reduction, albeit to a slightly lesser extent, from 4.65 mm at baseline to 4.44 mm at six weeks and 4.25 mm at 12 weeks.

These results align with previous studies that have demonstrated the efficacy of Propolis in reducing PPD [23, 24]. Propolis, known for its anti-inflammatory and antibacterial properties, has been investigated for its potential in periodontal therapy [25]. Several studies have reported reductions in PPD when using Propolis-based products, attributed to its ability to inhibit inflammatory processes and microbial growth within periodontal pockets [7, 26].

### Bleeding on probing (BOP)

Bleeding on Probing (BOP) is a crucial clinical parameter in periodontal evaluation, reflecting the inflammatory status of periodontal tissues. The study found that BOP significantly decreased in both the Propolis and chlorhexidine groups, with a slightly greater reduction observed in the Propolis group. In the Propolis group, BOP decreased from a mean value of 77.20 to 46.30 at six weeks and 14.60 at 12 weeks. In the chlorhexidine group, BOP reduced from 77.30 to 49.60 at six weeks and 22.80 at 12 weeks.

The results of this study align with previous research findings on the efficacy of propolis as a natural therapeutic agent in the treatment of periodontal disease [22, 27]. Propolis has been known for its antimicrobial, anti-inflammatory, and antioxidant properties, which can contribute significantly to the healing process after mechanical periodontal therapy [28]. This might explain the more pronounced reduction in BOP observed in the propolis group.

### Clinical attachment loss (CAL)

Clinical Attachment Loss (CAL) is another essential parameter for assessing periodontal health. The study’s results reveal a significant reduction in CAL for both groups, indicating improved attachment between the teeth and surrounding tissues. In the Propolis group, CAL decreased from 4.45 mm at baseline to 4.15 mm at six weeks and 3.77 mm at 12 weeks. The chlorhexidine group showed a reduction from a baseline value of 4.80 mm to 4.50 mm at six weeks and 4.19 mm at 12 weeks.

These findings corroborate previous research indicating the efficacy of Propolis in reducing CAL [29]. Propolis, with its anti-inflammatory and tissue regenerative properties, has shown promise in promoting attachment between the periodontal tissues and teeth [17]. Studies have suggested that Propolis can stimulate fibroblast activity and collagen synthesis, contributing to improvements in CAL [24, 30].

On the other hand, chlorhexidine, a widely accepted antiseptic in dentistry, also showed a considerable reduction in PPD, CAL, and BOP, consistent with the existing literature [11]. The mechanism of action mainly lies in its ability to cause bacterial cell death by disrupting the cell membrane [9].

However, this study observed a higher degree of reduction in the aforementioned clinical parameters in the Propolis group compared to the chlorhexidine group. These findings can be attributed to the additional anti-inflammatory and antioxidant properties of Propolis that can provide an added benefit in periodontal healing, a hypothesis supported by some recent studies [20, 31].

Sukhmawati (2021) conducted a study on six participants, and in each participant, two different sites of periodontal pockets were chosen. Group A received 10% propolis after the curettage, and Group B was given 1% tetracycline after curettage. PI, PPD, BOP, and concentration of IL-1β were assessed at baseline and day 21. Significant reductions in IL-1β and clinical parameters such as PPD and BOP were recorded, strengthening the findings of our study [24].

Propolis was administered as an adjunct to chlorhexidine in two studies, and the results of both groups were comparable, and Propolis reduced the periodontal index equally with chlorhexidine [29, 32]. Propolis mouthwash was also tested against placebo after mechanical debridement of the pockets. The final visit significantly improved PPD, CAL, and BOP compared to placebo [26].

The results of this study align with previous research indicating the effectiveness of Propolis in the treatment of periodontal disease [33, 34]. The reduction in PPD, CAL, and BOP in the Propolis group is in line with earlier investigations that have reported similar outcomes [35, 36]. This consistency in findings suggests that Propolis may be a valuable adjunct in periodontal therapy.

It is important to note that the present study contributes to the existing body of evidence by providing a well-structured clinical trial with rigorous methodology. By conducting a double-blind, randomized, controlled trial, the authors have addressed potential sources of bias and increased the reliability of their findings. This study has several strengths, including the large sample size and full mouth examinations of all patients. Additionally, all patients were examined by the same examiner, trained to ensure consistency in results. Although measures have been taken to avoid missing follow-up, clinical trials are always limited by participant exclusions and decreases in sample size that researchers cannot control. Other biomarkers in addition to clinical parameters assist in determining the superiority of Propolis over traditional medicaments. For this purpose, salivary biomarker Neopterin was measured in saliva of patients in periodontitis, which will be discussed in Part 2 of this manuscript.

Despite these promising findings, it is crucial to consider the limitations of the study, such as the short follow-up period. Future research with longer follow-up periods can provide more concrete evidence on the comparative efficacy of Propolis and chlorhexidine mouthwashes in the treatment of chronic periodontitis, especially in perimenopausal women. Future studies should offer incentives to participants to improve compliance and make it easier to contact them when evaluations are required.

Based on the findings of the present study, the following recommendations can be made for future research and clinical practice. Considering the chronic nature of periodontitis and its management, future studies should incorporate extended follow-up periods. This would provide more robust data on the long-term efficacy and safety compared to chlorhexidine. The current study focused on perimenopausal women. Future research could consider a more diverse population, including males and females across different age groups, to examine the effects of propolis and chlorhexidine mouthwashes on a broader demographic. As both types of mouthwash demonstrated beneficial effects, their combined use might be worth investigating to understand if simultaneous use can offer synergistic benefits in the treatment of chronic periodontitis. Further research is warranted to understand the exact mechanism through which propolis provides an added advantage in periodontal healing. This could lead to the formulation of more effective therapeutic agents. Given the superior effect of propolis, a cost-effectiveness analysis could be undertaken to examine the potential economic benefits of using propolis over chlorhexidine, considering factors such as production cost, patient compliance, and side effects. In addition to clinical parameters, future studies may incorporate patient-reported outcome measures such as comfort, taste preference, and overall satisfaction to provide a comprehensive evaluation of the effectiveness of mouthwashes. Lastly, based on the results of the current study, it may be recommended that clinicians consider the use of propolis mouthwash as an adjunct to mechanical therapy in perimenopausal women with chronic periodontitis.

## Conclusion

In conclusion, the results of this study support the use of Propolis as a viable treatment option for chronic periodontitis. Both Propolis and chlorhexidine mouthwash had a favourable effect on improving the clinical parameters. However, Propolis mouthwash was more effective in resolving the PPD and reducing BOP. The outcome of the CAL treatment was identical in both groups. Propolis’s ability to reduce PPD, CAL, and BOP, as demonstrated in this research, adds to the growing body of evidence suggesting the potential of Propolis in periodontal therapy. Further research and longer-term studies are needed to explore the sustained effects of Propolis and its potential for broader clinical applications in periodontal care.

## Data Availability

The data that support the findings of this study are available from the corresponding author upon reasonable request.
